# Breaking the pain barrier: implantable intrathecal pump therapy as a game-changer in cancer pain management

**DOI:** 10.2478/raon-2025-0060

**Published:** 2025-12-16

**Authors:** Iztok Potocnik, Branka Strazisar, Helena Lenasi, Teodora Zupanc

**Affiliations:** 1 Department of Anesthesiology, Intensive Care and Pain Therapy, Institute of Oncology Ljubljana, Ljubljana, Slovenia; 2 Department of Anesthesiology and Reanimatology, Faculty of Medicine, University of Ljubljana, Ljubljana, Slovenia; 3 Institute of Physiology, Faculty of Medicine, University of Ljubljana, Ljubljana, Slovenia

**Keywords:** cancer pain, intrathecal drug delivery, implantable pumps, palliative care, opioid-sparing therapy

## Abstract

**Background:**

Chronic cancer pain, especially in advanced stages, remains a significant clinical challenge, often necessitating complex multimodal strategies. Although systemic opioids are standard therapy, many patients experience inadequate relief or adverse effects. Implantable intrathecal drug delivery systems (IDDS) have emerged as a promising alternative, enabling targeted analgesia with reduced opioid burden and improved quality of life. This narrative review summarizes current evidence on the clinical application, efficacy, safety, and cost-effectiveness of IDDS in cancer pain management. Literature sources include clinical trials, observational studies, health-economic evaluations, and international guidelines published between 2002 and 2023. A Slovenian case report is included, detailing the first national experience with IDDS implantation for refractory cancer pain. Clinical outcomes were assessed using the Visual Analogue Scale (VAS), European Organization for the Research and Treatment of Cancer Quality of Life Questionnaire (EORTC QLQ-C30), and the revised Edmonton Symptom Assessment System (r-ESAS).

**Conclusions:**

Findings from the literature confirm that intrathecal pumps provide substantial and sustained pain relief, often with a significant reduction in systemic opioid doses and associated side effects. Compared to conventional pharmacotherapy, intrathecal delivery is associated with improved patient-reported outcomes, fewer hospitalizations, and lower long-term healthcare costs. In the Slovenian case, VAS scores decreased from > 8 to 3 shortly after implantation, with parallel improvements in quality-of-life indices. IDDS represent a clinically effective and economically sustainable option for selected patients with complex cancer pain, particularly when systemic therapy proves insufficient. Their integration into multidisciplinary palliative care pathways supports personalized, safe, and compassionate treatment approaches. By combining an evidence-based overview with real-world national experience, this review underscores the therapeutic value of intrathecal drug delivery and calls for broader clinical awareness and future research.

## Introduction

The increased survival rate among cancer patients over recent decades has significantly transformed the clinical approach to cancer-related pain, shifting the primary objective from short-term symptom control to long-term management of chronic pain conditions.^1,2^ As cancer increasingly becomes a chronic illness for many, the burden of persistent pain affects a substantial proportion of patients, with direct implications for physical functioning, psychological well-being, and overall quality of life. This clinical evolution necessitates treatment modalities that not only ensure sustained analgesia but also carry a minimal side-effect profile and support the patient’s autonomy and daily functioning.^3^

In this context, considerable attention has been directed toward optimizing drug delivery systems to achieve more effective and tolerable pain control. Alternative routes of administration have been widely explored to enhance the therapeutic ratio of analgesics, aiming to maintain efficacy while reducing systemic toxicity and improving patient comfort.^4,5^ These strategies are especially critical in advanced or refractory cases where conventional oral or transdermal analgesics fail to provide sufficient relief or are associated with intolerable side effects.

For patients with intractable or complex pain syndromes, medications have traditionally been administered via subcutaneous infusion or directly into the central nervous system, either into the subarachnoid or epidural space. These approaches involve the use of specialized catheters and external infusion pumps designed to deliver analgesics continuously or intermittently.^6^ However, despite their analgesic effectiveness, external pump systems often pose logistical challenges: frequent replacement of drug mixtures is typically required due to high infusion volumes, necessitating repeated visits to the pharmacy and healthcare providers – on average every 5 to 7 days – which can impose significant burdens on patients and caregivers alike.^7,8^

In response to these limitations, implantable intrathecal pump systems have been introduced in many developed countries, including the United States, Canada, and nations in Western Europe.^9^ These systems involve the surgical implantation of a programmable pump connected to a catheter that delivers medication directly into the intrathecal space. The delivery rate can be precisely adjusted using a physician-controlled programmer, allowing individualized treatment regimens.^10^ Evidence supports the use of intrathecal analgesia via implantable pumps as a safe and effective modality for managing chronic cancer pain, particularly in patients with high opioid requirements or intolerance to systemic routes.^11^

The advantages of this method over systemic and epidural analgesia are considerable. These include improved pain control with lower total drug dosages, a reduction in systemic and neurotoxic side effects, fewer complications related to catheter management, lower infection rates, and decreased need for maintenance procedures.^12^ Importantly, intrathecal delivery also reduces systemic opioid exposure, which may help mitigate the risk of opioid-induced tumour progression, a phenomenon that has been associated with activation of μ-opioid receptors present on certain tumour cells.^13,14^

### Cancer pain

Pain remains one of the most prevalent and distressing symptoms experienced by individuals with cancer and is a leading cause of suffering in this population. Its incidence and intensity tend to increase as the disease advances, affecting an estimated 60% to 90% of patients in the later stages of illness.^1,2^ This high prevalence reflects the complex and multifactorial nature of cancer pain, which often results from a combination of direct tumour-related effects, treatment-induced injuries, and systemic consequences of malignancy. Effective management of cancer pain is not only essential for alleviating physical discomfort but is also a cornerstone of preserving patient dignity, emotional well-being, and overall quality of life.^1,2^

Cancer pain is pathophysiologically heterogeneous, encompassing a broad spectrum of mechanisms that frequently coexist in the same patient. Nociceptive pain, one of the primary components, arises from activation of pain receptors due to direct tissue injury. This may result from tumor infiltration into bones, soft tissues, visceral organs or tissue damage, caused by surgery or radiation.^1,2^ In contrast, neuropathic pain stems from damage or dysfunction of the peripheral or central nervous system, often due to tumour compression of neural structures, neurotoxic effects of chemotherapy, or post-radiation nerve injury. Notably, many cancer patients experience mixed pain – a complex combination of nociceptive and neuropathic components – which complicates both diagnostic clarity and therapeutic planning.

Beyond these direct mechanisms, several additional factors modulate the perception and intensity of cancer pain. Psychological distress – including anxiety, depression, existential suffering, and anticipatory fear – has a well-documented capacity to amplify pain perception and reduce patients’ coping ability. Furthermore, chronic inflammation, paraneoplastic syndromes, metabolic derangements, and immunological changes associated with malignancy may further sensitize pain pathways or lower the threshold for nociception.^1,2^

The inherently dynamic and evolving nature of cancer pain necessitates a personalized, multidisciplinary approach to assessment and treatment. Comprehensive pain management must integrate not only pharmacological interventions tailored to the underlying pathophysiology but also address psychosocial and spiritual dimensions of the patient’s experience. Early recognition and proactive treatment are therefore critical to prevent pain chronification, maintain functional capacity, and enhance the overall trajectory of care in oncology patients.^1,2^

### Recommendations on cancer pain treatment

Managing chronic refractory cancer pain remains one of the most persistent and complex challenges in oncology and palliative care. Unlike many other clinical symptoms, the severity of cancer-related pain often does not exhibit a straightforward correlation with tumour burden or anatomical progression, making both assessment and treatment highly individualized and unpredictable. When left inadequately controlled, cancer pain not only impairs functional capacity and emotional resilience, but also negatively affects adherence to anticancer treatments, potentially compromising therapeutic outcomes and overall prognosis.

**FIGURE 1.**
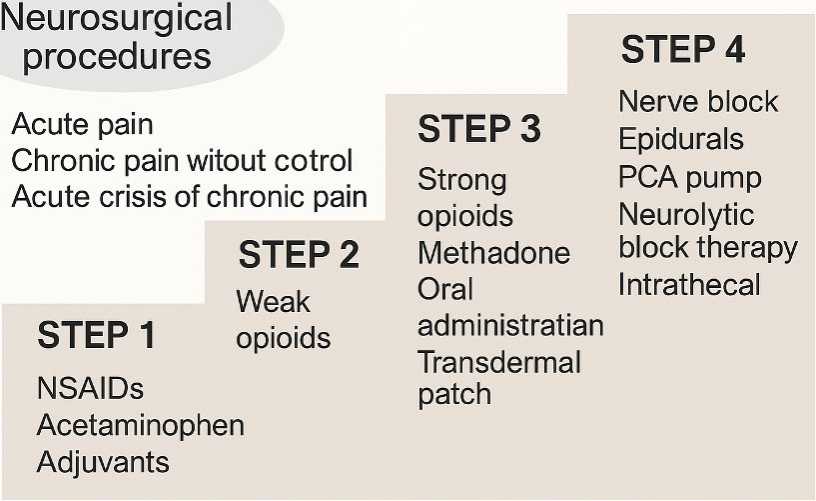
Updated WHO analgesic ladder. Stepwise model of cancer pain management, progressing from NSAIDs to intrathecal and neurosurgical interventions. IDDS = intrathecal drug delivery system; NRS = Numerical Rating Scale; NSAIDs = nonsteroidal anti-inflammatory drugs; PCA = patient-controlled analgesia; VAS = Visual Analogue Scale; WHO = World Health Organization

The World Health Organization (WHO) established a foundational framework for cancer pain management with its three-step analgesic ladder, which proposes a progressive escalation of pharmacological therapy based on pain severity: beginning with non-opioid analgesics, advancing to weak opioids for moderate pain, and strong opioids for severe pain.^1,15^ More recently, this model has been conceptually expanded with the addition of a fourth step, encompassing interventional regional techniques such as peripheral nerve blocks, neuraxial analgesia, and implanted catheters (Figure 1). These interventions are intended to supplement each level of pharmacological treatment, particularly in cases where conventional therapies fail or are poorly tolerated.

However, despite the availability of this structured framework and its broad endorsement across international guidelines, a substantial proportion of cancer patients experience insufficient pain control when treated solely according to the WHO ladder.^3^ Multimodal and individualized pain management strategies – integrating pharmacological, interventional, and psychosocial modalities are increasingly recognized as the gold standard, as reflected in most European and global recommendations.^2,3^ Indeed, clinical studies have revealed that after four weeks of opioid-based treatment, only about 25% of cancer patients report satisfactory pain relief (VAS < 4).^4^ Moreover, systemic administration of opioids is frequently accompanied by debilitating adverse effects, including nausea, vomiting, constipation, confusion, and excessive sedation, all of which can limit both adherence and tolerability.^5^

A comprehensive and personalized assessment of pain is essential for effective management. This includes characterizing the intensity, location, temporal pattern, and underlying mechanisms of pain whether nociceptive, neuropathic, or mixed. Standardized assessment tools such as the VAS or the Numerical Rating Scale (NRS) allow quantifiable tracking of symptom severity over time. For mild pain, first-line agents typically include non-opioid analgesics such as acetaminophen or non-steroidal anti-inflammatory drugs (NSAIDs). For moderate to severe pain, strong opioids – such as morphine, oxycodone, or transdermal fentanyl – are indicated, in accordance with the WHO ladder (Table 1). In cases involving neuropathic components or inflammatory pain, adjuvant agents such as antidepressants (e.g., duloxetine), anticonvulsants (e.g., gabapentin), or corticosteroids are commonly employed to enhance analgesic efficacy.

**Table 1.** Pharmacological treatment according to the WHO analgesic ladder

Mild pain	Moderate pain	Severe pain
NSAID Paracetamol Metamizole	Tramadol Codeine	Morphine Fentanyl Methadone Oxycodone Hydromorphone Buprenorphine Tapentadol

1 NSAIDs = nonsteroidal anti-inflammatory drugs; WHO = World Health Organization

For patients with refractory or complex pain syndromes, interventional strategies become necessary. These may include neuraxial techniques such as epidural or intrathecal administration of analgesics, peripheral nerve blocks, or neuromodulatory interventions like spinal cord stimulation. In parallel, the integration of psychological support, structured palliative care involvement, and family-based interventions is vital to address the multifaceted emotional and existential dimensions of cancer pain.

According to the European Society for Medical Oncology (ESMO), the presence of persistent and often disabling pain – particularly in advanced disease – demands a systematic, patient-centered management approach. As survival rates improve due to advances in oncological therapies, an increasing number of patients are living with chronic pain resulting from the disease itself, its treatments, or both. Despite access to clinical guidelines and pharmacological resources, undertreatment remains widespread, due to factors including underreporting of symptoms, inadequate assessment, opioid hesitancy among clinicians, and systemic barriers to care.^16,15^

ESMO guidelines therefore strongly advocate early and proactive involvement of palliative care teams, even during the active phase of cancer treatment. This interdisciplinary model promotes regular reassessment of pain control, titration of analgesics, and aggressive management of side effects such as opioid-induced constipation and sedation – measures that significantly enhance both compliance and therapeutic outcomes. Furthermore, the guidelines highlight the critical importance of ongoing education and training for healthcare professionals to overcome clinical inertia and misconceptions surrounding opioid prescribing.^16^

Ultimately, the goal of cancer pain management extends beyond mere symptom suppression. It encompasses restoration of dignity, autonomy, and engagement in life, regardless of prognosis. Tailoring analgesic strategies to individual needs – while addressing the physical, emotional, and psychosocial domains of suffering – is fundamental to high-quality oncological and palliative care.^1,2,16^

### Breakthrough pain

Managing breakthrough pain (BTP) in cancer patients presents a distinct and complex therapeutic challenge. Despite increasing clinical recognition over the past decades, persistent inconsistencies in the definition and classification of BTP continue to hinder timely diagnosis and appropriate intervention.^16^ As a result, many patients endure frequent and intense pain exacerbations, often for prolonged periods and with inadequate symptom relief, even after initial identification of the condition.^16^

Breakthrough pain, first formally characterized in the 1990s, refers to transient episodes of severe pain that occur despite otherwise controlled baseline pain achieved through around-the-clock opioid therapy.^17^ These episodes typically represent acute exacerbations superimposed on a stable analgesic regimen and are frequently unpredictable in onset. Epidemiological studies report that approximately 60% of patients with advanced cancer experience severe breakthrough pain (VAS > 7), and a further 30% report episodes of moderate intensity (VAS > 5).^6,7^ The high prevalence and severity of BTP underscore its significant impact on patient well-being, daily functioning, and overall quality of life.

According to Rudowska’s 2012 definition, breakthrough pain is characterized by sudden-onset, high-intensity pain episodes – often described as “flares” – which generally last around 30 minutes and occur in the same anatomical region as the patient’s background pain.^19,18^ These episodes are typically rated between 7 and 10 on the VAS, indicating severe intensity. Importantly, BTP is not merely a transient discomfort but a profound clinical event that significantly exacerbates physical suffering and psychological distress. Delays in treatment initiation or insufficient rescue medication not only prolong pain duration but also impair functional status, reduce treatment adherence, and negatively influence the overall cancer care experience.^7,8,9^

Despite effective control of background pain, breakthrough pain remains common, with up to 60% of patients in advanced stages of cancer reporting such episodes.^10,11^ The episodic and often unpredictable nature of BTP necessitates rapid-onset, short-acting analgesics, tailored to the individual characteristics of each pain episode. Prompt recognition and intervention are therefore paramount. In clinical practice, rescue medications – usually fast-acting opioids – are administered at the onset of a breakthrough episode to restore adequate analgesia. The choice of agent and route of administration depends on the anticipated onset, intensity, and duration of pain. Sublingual, buccal, or intranasal fentanyl preparations are commonly preferred for their rapid absorption and onset of action, making them suitable for short-lived, severe pain episodes that demand immediate relief.^12,15,16,17^ For episodes with a slower onset or prolonged duration, oral morphine may be considered, although it is generally less effective for managing the sudden peaks in pain characteristic of BTP. Regardless of the pharmacological agent used, the timing of administration is critical; delays can substantially reduce efficacy and compound the patient’s suffering.

Overall, the management of breakthrough pain requires not only appropriate pharmacological strategies but also ongoing patient education, routine monitoring, and anticipatory guidance. Patients and caregivers should be equipped to recognize early signs of BTP and initiate treatment promptly, ideally within minutes of onset. Integrating breakthrough pain protocols into comprehensive cancer pain management plans enhances therapeutic outcomes and aligns with the overarching goals of maintaining dignity, autonomy, and comfort throughout the cancer trajectory.^6–12,15–17^

### Multimodal approach to cancer pain management

The management of chronic refractory cancer pain remains one of the most intricate and demanding aspects of oncological and palliative care. A central challenge lies in the fact that pain severity in cancer patients often does not correlate linearly with objective indicators such as tumour size or anatomical progression. This discordance complicates clinical assessment and underscores the need for individualized and dynamic treatment strategies. Inadequate control of cancer pain – whether persistent or episodic – not only compromises physical functioning and quality of life but also contributes to psychological morbidity, reduced adherence to anticancer therapies, and, ultimately, poorer clinical outcomes.^1,2^

Contemporary evidence and international guidelines strongly support the use of multimodal approaches for the management of both persistent and breakthrough cancer pain.^1,2,3^ These strategies aim to address the complex and multifactorial nature of cancer-related pain by combining various therapeutic modalities, each targeting different components of the pain pathway. Effective multimodal pain management is not a one-size-fits-all approach; rather, it requires tailoring interventions to the patient’s individual clinical profile, taking into account the underlying pathophysiology, psychosocial context, and treatment goals.^16^

Central to the multimodal paradigm is the integration of neuromodulatory techniques – particularly the intrathecal or epidural administration of analgesics – which enable targeted drug delivery directly into the central nervous system.^4,5^ These methods offer potent analgesia with substantially reduced systemic opioid exposure and are especially valuable in patients with refractory pain who have not responded adequately to non-invasive or systemic therapies. The use of implantable pump systems for continuous intrathecal delivery has demonstrated sustained efficacy in pain relief, reduced side effects, and improved patient autonomy and quality of life.^6,9,18^

In parallel, less invasive and complementary approaches such as acupuncture, physical therapy, psychological support, and cognitive-behavioural interventions play a crucial role in enhancing analgesic outcomes and supporting the emotional and functional resilience of patients. The synergistic effect of combining pharmacological, interventional, and supportive therapies allows for improved symptom control while minimizing reliance on any single modality.^3^

The selection of an appropriate neuromodulatory technique must be guided by a comprehensive assessment of the patient’s clinical condition, including pain characteristics, comorbidities, functional status, psychosocial context, and patient preferences.^18^ Factors such as age, cancer type and stage, history of response to previous pain treatments, and anticipated prognosis all inform the therapeutic plan. Importantly, a thorough evaluation should extend beyond somatic symptoms to include emotional, cognitive, and spiritual domains, recognizing the total pain experience as defined in palliative medicine.

The multimodal approach places strong emphasis on personalized care, ongoing reassessment, and interdisciplinary collaboration. Pain management is most effective when delivered within a coordinated framework involving oncologists, palliative care specialists, anesthesiologists, psychologists, physiotherapists, and nursing staff. This integrative model ensures that treatment remains aligned with evolving disease status and patient priorities. Regular review of therapeutic efficacy, tolerability, and emerging needs allows for timely adjustments and optimal resource allocation.^2,7^

Ultimately, the goal of multimodal cancer pain management is not merely to reduce pain intensity but to enhance the overall well-being, dignity, and quality of life of patients at every stage of the disease trajectory. When tailored to the unique and dynamic needs of the individual, multimodal strategies can transform the experience of pain from a source of suffering to a domain of compassionate and effective clinical care.^1,3^

### Invasive techniques for cancer pain treatment

Invasive techniques constitute a vital component of cancer pain management, particularly for patients who are unable to tolerate oral medications due to side effects such as persistent nausea, vomiting, or dysphagia, as well as for those suffering from neuropathic pain unresponsive to systemic therapies. These methods provide a targeted, often more effective approach to analgesia when conventional pharmacological strategies prove inadequate. Among these, intrathecal drug administration is widely recognized for its superior efficacy and safety profile, offering several advantages over other invasive modalities, including the use of significantly lower analgesic doses, reduced systemic toxicity, fewer side effects, lower risk of infection, and improved overall pain control.^5,6,18^ Additionally, minimizing systemic opioid use through intrathecal delivery may help attenuate opioid-related adverse effects and mitigate concerns about potential cancer progression driven by opioid receptor activation.

Beyond intrathecal therapy, a range of interventional pain management techniques plays a crucial role in addressing refractory cancer pain. These include nerve blocks, spinal cord stimulation (SCS), and neurolytic procedures, each offering distinct benefits based on pain type and anatomical location. Nerve blocks, administered using local anesthetics or neurolytic agents such as alcohol or phenol, can provide profound and lasting relief for well-localized cancer pain, such as that associated with pancreatic or pelvic malignancies. For instance, celiac plexus blocks are commonly employed in upper abdominal cancers, while superior hypogastric plexus blocks are indicated for pelvic tumours.^5,6,18^

Spinal cord stimulation, involving the implantation of an electrode system to deliver electrical impulses to the dorsal columns of the spinal cord, is an effective treatment for selected cases of chronic, refractory neuropathic pain, particularly when pharmacological options have been exhausted or poorly tolerated. It modulates pain signal transmission without the systemic burdens of opioid therapy and can significantly improve quality of life in patients with intractable pain syndromes.

Neurolytic procedures, aimed at ablating specific nerve pathways, are typically used for visceral pain in advanced cancer stages. Techniques such as celiac plexus or hypogastric nerve ablation are particularly effective for deep-seated abdominal or pelvic pain, offering sustained relief that may last weeks to months, thereby reducing analgesic burden and improving patient comfort.^5,6,18^

The decision to implement invasive techniques must be based on a comprehensive, multidisciplinary evaluation, considering factors such as the nature and mechanism of pain, expected prognosis, existing comorbidities, patient preferences, and overall functional and psychological status. Pre-procedural assessments, including imaging, functional testing, and temporary diagnostic blocks, are essential to predict treatment response and mitigate risks. The primary aim is not merely analgesia but the preservation of functionality, enhancement of quality of life, and support for the patient’s emotional and existential well-being.^18^

Recent technological advances, including image-guided interventions and programmable intrathecal pump systems, have further refined the precision and efficacy of invasive pain control. These innovations allow for tailored targeting of pain generators and permit dynamic modulation of analgesic delivery in response to clinical changes. Integration of invasive procedures into a broader multimodal pain management framework ensures a holistic, patient-centered approach that concurrently addresses the physical, psychological, and spiritual dimensions of suffering.^5,6,18,19^

While invasive techniques are generally reserved for patients with complex, refractory, or advanced-stage pain, they have emerged as cornerstones of modern cancer pain therapy. Their success exemplifies the value of individualized, evidence-based intervention, as well as the indispensable role of ongoing research, innovation, and interdisciplinary collaboration in optimizing outcomes for cancer patients confronting intractable pain.^5,6,18,19^

### Implantable intrathecal pumps

Implantable intrathecal pump systems represent a pivotal advancement in the field of cancer pain management, offering targeted, sustained analgesia for patients with refractory or complex pain syndromes. First introduced in Western Europe in 1984, these devices have progressively gained acceptance as an integral component of neuro-modulatory pain therapy. In Slovenia, their use was initially established in 2001 for the treatment of spasticity and was expanded to include cancer-related analgesia in January 2024 at the Institute of Oncology, Ljubljana.^20^ These systems are now recognized as a safe and effective modality for delivering analgesics directly into the intrathecal space, enabling significant reductions in drug dosage compared to systemic or parenteral administration, with a corresponding decrease in adverse effects.^20^

The implantation procedure involves the surgical insertion of a catheter into the intrathecal space, typically under general anesthesia, and the subcutaneous placement of the pump reservoir. The procedure is generally well tolerated and is performed by specialized neurosurgeons or anesthesiologists with expertise in interventional pain management. Once implanted, the system allows precise control over drug delivery, with programmable infusion parameters that can be tailored to the patient’s evolving clinical needs. Moreover, many devices offer patient-controlled bolus functionality, which enables the patient to self-administer additional doses in the event of breakthrough pain, thus enhancing autonomy and responsiveness of care.

A major clinical advantage of intrathecal pump therapy is the significant reduction in systemic opioid-related side effects, particularly gastrointestinal toxicity. For example, constipation rates in patients treated with intrathecal opioids are markedly lower (7%) compared to those receiving systemic opioids (43%).^21^ The reduced incidence of sedation, nausea, and cognitive impairment further supports the preference for intrathecal delivery in appropriate candidates. Additionally, due to the high concentration and low volume of medication required, the frequency of pump refills is substantially reduced, decreasing the burden on patients and caregivers and improving overall quality of life.^21^

The efficacy of intrathecal analgesia in cancer pain has been supported by clinical research. Studies conducted by Dupoiron *et al*. have demonstrated the capacity of these systems to maintain stable and long-lasting pain control while minimizing systemic opioid use and associated toxicities, thereby reinforcing their role in individualized pain management protocols.^22^ Likewise, Likar *et al*. have shown that incorporating intrathecal pumps into palliative care pathways significantly improves patient outcomes in advanced cancer, where conventional pharmacotherapy frequently fails to provide adequate relief.^23^

Technological advancements have further enhanced the utility of intrathecal pump systems. Programmable pumps now allow real-time adjustment of infusion rates and regimens, offering flexibility to respond to fluctuating pain intensities in patients with complex or rapidly changing pain profiles. In addition, multidrug infusions – such as combinations of opioids with local anesthetics or adjuvants like clonidine – provide synergistic analgesic effects, allowing for more comprehensive pain modulation while minimizing single-agent toxicity.^24^

Long-term management of intrathecal systems requires ongoing interdisciplinary collaboration among oncologists, anesthesiologists, palliative care teams, and nursing staff. Regular monitoring, periodic refills, and prompt troubleshooting are essential for maintaining safety and efficacy. Despite the need for consistent follow-up, patients typically report substantial improvements in quality of life, increased mobility, and reduced emotional distress – benefits that justify the procedural and maintenance demands associated with this modality.

In conclusion, implantable intrathecal pumps are a cornerstone intervention within the multimodal framework of cancer pain management. Their effectiveness, adaptability, and patientcentered nature make them an indispensable tool, particularly in advanced-stage disease. As emphasized by Dupoiron^22^ and Likar.^23^, continued clinical research is essential for refining the indications, optimizing protocols, and expanding the accessibility of these devices to broader patient populations.

### Cost-effectiveness of intrathecal implantable pumps

Intrathecal implantable pumps represent a sophisticated and clinically effective solution for delivering targeted analgesia in patients with severe, refractory cancer pain, particularly within palliative care and oncology settings. Although the initial costs associated with surgical implantation and device acquisition are relatively high, a growing body of evidence suggests that intrathecal therapy is cost-effective over the long term, primarily due to its ability to reduce medication requirements, limit treatment-related complications, and decrease healthcare resource utilization.

Intrathecal drug delivery permits the direct administration of opioids and adjuvants into the cerebrospinal fluid, bypassing the systemic circulation and enabling the use of substantially lower drug doses compared to oral or parenteral routes.^24^ This pharmacokinetic advantage results in lower cumulative costs for analgesics, especially in patients requiring high-dose opioid therapy over extended periods. Importantly, by reducing systemic exposure, intrathecal administration also significantly decreases the incidence and severity of opioid-related side effects such as constipation, nausea, sedation, and cognitive dysfunction, which are common drivers of additional healthcare interventions, hospital admissions, and patient distress.

Patients receiving intrathecal therapy demonstrate improved symptom control, which translates into fewer emergency department visits, unplanned hospitalizations, and reduced need for supportive care associated with uncontrolled pain.^25^ These factors not only alleviate the burden on healthcare systems but also contribute to a higher quality of life and functional preservation for patients in advanced stages of disease. The capacity of intrathecal pumps to stabilize complex pain syndromes with minimal systemic burden makes them particularly valuable in resource-sensitive environments focused on optimizing both clinical outcomes and economic sustainability.

While the upfront investment in intrathecal pump therapy – including surgical placement, pump programming, and ongoing maintenance – may appear cost-prohibitive at first glance, multiple health economic evaluations have demonstrated long-term financial advantages. A landmark study by Rauck *et al*. showed that patients treated with intrathecal therapy incurred lower cumulative healthcare costs after the first year of treatment when compared with those managed with systemic opioids. These savings were attributed to reduced drug expenditures, fewer side-effect-related interventions, and a marked decrease in hospital resource consumption.^26,27^

In addition to economic benefits, the clinical efficiency of intrathecal systems justifies their integration into multimodal pain management frameworks, particularly for patients with high analgesic requirements, complex pharmacological profiles, or contraindications to systemic therapy. The long-term sustainability of these systems is further supported by advances in pump technology, which allow for programmable dose modulation, extended refill intervals, and combination drug infusions – all of which improve therapeutic precision and patient satisfaction.^24^

In summary, although intrathecal implantable pumps require initial capital investment, their ability to reduce ongoing treatment costs, improve patient outcomes, and decrease healthcare utilization supports their use as a cost-effective solution in the comprehensive management of cancer pain. As healthcare systems increasingly prioritize value-based care, intrathecal therapy stands out as a compelling option for addressing the dual imperatives of clinical efficacy and economic efficiency in advanced oncologic pain management.^24–27^

### Decrease in opioid consumption with intrathecal administration

A fundamental advantage of intrathecal opioid administration lies in its ability to achieve potent analgesia with a drastically lower total opioid dose compared to systemic delivery – 100-fold or even 300-fold lower compared with oral administration.^24,26,27^ This approach takes advantage of direct access to the opioid receptors located in the dorsal horn of the spinal cord, allowing for highly localized receptor activation. By bypassing first-pass hepatic metabolism and systemic distribution, intrathecal administration ensures maximal pharmacodynamic efficiency at the site of action while minimizing peripheral drug exposure. This substantial reduction in required opioid quantity translates into a marked decline in the incidence of opioid-related side effects, such as sedation, nausea, vomiting, constipation, urinary retention, and cognitive impairment, which are often dosedependent and can severely limit the tolerability of systemic analgesic regimens.^28^

In addition to improving tolerability, lower opioid exposure also reduces the likelihood of developing opioid tolerance, which necessitates escalating doses over time and contributes to a vicious cycle of increasing toxicity and diminishing efficacy. Intrathecal therapy helps interrupt this cycle by stabilizing analgesic requirements and delaying or preventing opioid-induced hyperalgesia – a paradoxical condition in which opioids worsen rather than relieve pain. Furthermore, by minimizing systemic opioid load, intrathecal administrationmay lower the risk of physical dependence and iatrogenic addiction, especially in patients requiring long-term therapy for chronic cancer pain or palliative indications.^29^

The reduction in opioid dose made possible through intrathecal administration not only enhances clinical safety and effectiveness but also contributes to cost savings by decreasing the need for adjunctive medications used to manage side effects and by reducing hospitalization rates related to opioid toxicity. The ability to deliver precisely titrated, low-dose opioid regimens tailored to patient needs makes intrathecal therapy a particularly attractive modality in complex or refractory pain syndromes.

In conclusion, intrathecal opioid delivery provides a targeted, efficient, and safer alternative to systemic opioid therapy. Its capacity to drastically reduce opioid requirements while maintaining effective analgesia supports its expanding role in the long-term management of severe cancer pain, particularly in patients for whom systemic therapy is no longer viable or tolerable.^24,26–29^

### Our experiences with implantable intrathecal pumps

In Slovenia, three implantable intrathecal pumps have been successfully utilized to date. Here, we present our first case experience.

The patient was a 71-year-old female diagnosed with metastatic ocular malignant melanoma and inoperable urothelial carcinoma. She suffered from severe refractory nociceptive and neuropathic pain, significant side effects from high-dose opioid therapy, and a markedly reduced quality of life. Her pain was attributed to osteolytic metastases in the spine, radiating to the abdomen, lower extremities, and neurogenic bladder. Despite a multimodal pain management approach – including high-dose opioids, non-opioid analgesics, neuropathic agents, and adjuvants administered via various routes such as subcutaneous injections – the pain remained intractable. Palliative radiotherapy to metastatic sites was also provided.

Numerous modifications to her analgesic regimen yielded no significant relief, and her suffering became unbearable. Prior to intrathecal pump implantation, the patient’s regimen included a buprenorphine transdermal patch at 105 μg/hour, oral morphine 40 mg up to four times daily for breakthrough pain, paracetamol 1000 mg every 8 hours, and mirtazapine 30 mg. Despite this, her pain intensity remained severe (VAS > 8), predominantly localized to the lower abdomen and pelvic region, with mild radiation to both lower limbs. The pain was mixed in nature – somatic, visceral, and neuropathic. She also reported burning sensations during urination and frequent urinary urgency with minimal urine output.

The implantable intrathecal pump was inserted under general anesthesia by skilled neurosurgeons at Celje General Hospital. Initially, the pump was programmed to deliver 1 mg/day of morphine and 6 mg/day of bupivacaine intrathecally. Within three days, the patient experienced substantial pain reduction, with her VAS score decreasing to 3. For breakthrough pain, sublingual fentanyl was administered alongside continued peripheral analgesics. As the disease progressed, intrathecal doses were gradually increased; clonidine (65 μg/day) was added, and morphine and bupivacaine doses were titrated to 2.6 mg/day and 19.5 mg/day, respectively.

Pain and quality of life were assessed using the VAS score, the EORTC QLQ-C30, and the revised Edmonton Symptom Assessment System.^30,31,32^

- **EORTC QLrQ-C30** is a validated multidimensional questionnaire designed to assess quality of life in cancer patients. It evaluates functional domains (physical, role, emotional, cognitive, social), symptoms (fatigue, nausea/vomiting, pain, insomnia, appetite loss, constipation), and global health status/quality of life. Higher functional scores indicate better functioning; higher symptom scores indicate greater symptom burden. Higher global health status scores represent better overall quality of life and health.^31^
- **r-ESAS** is a widely used tool in palliative care for monitoring nine core symptoms on a numerical scale from 0 (none) to 10 (worst possible), plus an optional patient-defined symptom. It assesses pain, tiredness, nausea, depression, anxiety, drowsiness, appetite loss, well-being, and shortness of breath, enabling symptom tracking over time and assessment of treatment efficacy.^32^

Before pump implantation, the patient experienced opioid-related adverse effects such as constipation and dizziness due to high systemic opioid doses. Transitioning to intrathecal delivery allowed for a substantial opioid dose reduction and a corresponding decrease in side effects.

Three months post-implantation, significant improvements in quality of life were observed, reflected in both questionnaires (Tables 2 and 3). The EORTC QLQ-C30 showed improved symptom burden and functional domains. As expected, the global health status score did not improve due to disease progression and the patient’s subsequent death. Nevertheless, the primary goal – enhancing quality of life – was achieved. The r-ESAS results also indicated symptom improvement.

**Table 2.** Improvement in patients symptoms shown by EORTC QLQ-C30 questionnaire

Category	Parameter	Before (0-100)	After 3 months (0-100)
Symptoms	Pain	83.3	50
	Insomnia	100	33.3
	Appetite Loss	66.7	0
	Constipation	100	66.7
	Nausea and Vomiting	50	0
Functioning	Physical	73.3	80
	Role	66.7	100
	Emotional	0	75
	Cognitive	50	100
	**Social**	**50**	**100**

1 EORTC QLQ-C30= European Organization for the Research and Treatment of Cancer Quality of Life Questionnarire

**Table 3.** Improvement in patients symptoms shown by r-ESAS questionnaire. Each symptom is rated on a scale from 0 to 10, with a higher value indicating a more severe symptom

Parameter	Before (0-10)	After 3 months (0-10)
VAS (Pain)	5	0
Fatigue	3	0
Dizziness	3	0
Nausea	2	0
Inappetence	1	0
Dyspnoea	1	0
Depression	4	0
Anxiety	4	2
Overall Well-Being	4	2

1 r-ESAS = revised Edmonton Symptom Assessment System

The patient’s condition gradually deteriorated with disease progression, and she passed away six months after pump implantation.

## Discussion

Implantable intrathecal pumps represent a major advancement in the field of cancer pain management, offering precise, individualized, and long-term analgesia with a significantly improved side-effect profile compared to conventional systemic therapies. By delivering medication directly into the cerebrospinal fluid, these systems enable the use of substantially lower opioid doses, thus minimizing systemic exposure and associated toxicities such as sedation, nausea, and constipation. Furthermore, their programmable features and capacity for combination therapy allow for flexible, patient-centered pain control, even in cases involving complex or mixed pain mechanisms.

Globally, the adoption of implantable intrathecal pump therapy is reshaping standards in palliative oncology, offering a more effective and humane solution for both chronic baseline pain and breakthrough pain in patients with advanced disease. Our clinical experience – presented here in the form of a case report – demonstrates that intrathecal therapy can provide rapid, durable, and meaningful symptom relief, significantly enhancing quality of life in a patient suffering from refractory cancer pain. In this case, the introduction of intrathecal analgesia not only controlled previously intractable symptoms but also reduced opioid-related side effects and enabled improved functional capacity in the final months of life.

Beyond individual patient benefit, intrathecal pumps also offer systemic advantages for healthcare systems. Improved pain control reduces emergency department visits, hospital admissions, and the need for complex symptom management interventions, thereby lowering the burden on healthcare providers. Multiple cost-effectiveness studies have confirmed that, despite higher initial expenses for implantation and equipment, long-term use of intrathecal pumps results in reduced cumulative healthcare costs, particularly after the first year of therapy, owing to decreased drug use and resource utilization.

The successful implementation of the first intrathecal analgesia case in Slovenia marks a critical milestone in the integration of advanced pain management strategies within standard palliative care. This experience has laid the groundwork for broader clinical application and has prompted the development of a larger-scale study to systematically evaluate the clinical outcomes, safety, and economic impact of intrathecal pump therapy in cancer patients.

In conclusion, intrathecal therapy using implantable pumps should be regarded not only as an advanced technological intervention, but as a compassionate and evidence-based tool in the service of alleviating suffering. As our healthcare systems evolve to meet the growing demands of aging and oncologic populations, the integration of such therapies into routine care pathways is both a clinical imperative and an ethical responsibility.
